# Supplementary material for: The Role of BmTMED6 in Female Reproduction in Silkworm, Bombyx mori
Source: Insects. 2024 Feb 2;15(2):103. doi: 10.3390/insects15020103 (PMC10889480; doi:10.3390/insects15020103)
Supplement: Supplementary file 1 [file insects-15-00103-s001.zip › insects-2789133-Supplementary.pdf]

Table S1. Sequences of primers used in this study.

Primers	GenBank accession	Sequence (5′–3′)	Purpose
BmTMED6-BamHI-HindIII	OQ158769	F: CGCGGATCCGCGATGTTATCATCGTCAGCAGCACCAT<br>R: CCCAAGCTTGGGTCAATAAATATTGTTGTCTAACAG-CAGG	protein ex-pression
qBmTIF4	NM_001043911	F: TTCGTACTGGCTCTTCTCGT<br>R: CAAAGTTGATAGCAATTCCCT	qRT-PCR
qBmTMED6	OQ158769	F: GTTTATCATCGTCAGCAGCACC<br>R: CCGTCACCTCCTTTCAACACCT	
qBmDop2R2	XM_021347832.2	F: GAGCCAAATCATTCGCCAAGA<br>R: AAGAATGGTGCCCAGCAGAATAAG	
qBmDop2R1	XM_004925906.4	F: ACGCCCGACAGGAACTTTGA<br>R: GCCGCCCTTTGTTTCTTTG	
DsBmTMED6		F: TAATACGACTCACTATAGGGATGGCATTCAGGAC-GTGGC<br>R: TAATACGACTCACTATAGGGTCATATCCGAGTCTTT-GAGGAGTTG	dsRNA syn-thesis
DsEGFP		F: TAATACGACTCACTATAGGGAAAC-GGCCACAAGTTCAGCG<br>R: TAATACGACTCACTATAGGGGCTTCTCGTT-GGGGTCTTTG	
BmTMED6-BamHI-NotI	OQ158769	F: CGCGGATCCGCGATGCTATCTGCCTCTCTGTCA<br>R: TTGCGGCCGCAATCATATCCGAGTCTTTGAGGAGTTG	cellular over-expression
